# Comparison of Patient-collected and Lab Technician-collected Nasopharyngeal and Oropharyngeal Swabs for Detection of COVID-19 by RT-PCR

**DOI:** 10.30699/ijp.2020.127312.2387

**Published:** 2020-07-16

**Authors:** Alireza Abdollahi, Abbas shakoori, Hoda Khoshnevis, Mohammad Arabzadeh, Seyed Ali Dehghan Manshadi, Esmaeil Mohammadnejad, Dorsa Ghasemi, Maryam Safari Aboksari, Shaban Alizadeh, Vahid Mehrtash, Arezoo Eftekhar-javadi, Masoomeh Safaei

**Affiliations:** 1 *Department of Pathology, Imam Hospital Complex, Tehran University of Medical Sciences, Tehran, Iran*; 2 *Medical Genetic Ward, Imam Hospital Complex, Tehran University of Medical Sciences, Tehran, Iran*; 3 *Supervisor of Genetic Ward, Imam Hospital Complex, Tehran University of Medical Sciences, Tehran, Iran*; 4 *Laboratory Senior Technical Associate of Genetic Ward, Imam Hospital Complex, Tehran University of Medical Sciences, Tehran, Iran*; 5 *Department of Infectious Diseases and Tropical Medicine, School of Medicine, Tehran University of Medical Sciences, Tehran, Iran*; 6 *Department of Medical-Surgical Nursing, School of Nursing and Midwifery, Tehran University of Medical Sciences, Tehran, Iran*; 7 *Department of Pathology, School of Medicine, Tehran University of Medical Sciences, Tehran, Iran*; 8 *Hematology Department, Allied medical school, Tehran University of Medical Sciences, Tehran, Iran*

**Keywords:** COVID-19, Oropharynx, Nasopharynx, Real-time polymerase chain reaction, Specimen collection

## Abstract

**Background & Objective:**

A simple approach to prevent close contact in healthcare settings during the COVID-19 outbreak is to train patients to collect their own nasopharyngeal and oropharyngeal swabs and deliver them to medical laboratories to have them processed. The aim of our study was to compare lab technician- with patient- collected oropharyngeal and nasopharyngeal samples for detection of the coronavirus disease 2019 (COVID 19) using rapid real-time polymerase chain reaction (rRT-PCR).

**Methods:**

Fifty adult patients with flu-like symptoms and radiologic findings compatible with atypical pneumonia who were admitted to the infectious diseases ward of Imam Khomeini Hospital Complex, Tehran, Iran, with a clinical diagnosis of COVID-19 from February 28 to April 27 of 2020 were randomly selected and entered in our study. Two sets of naso- and oropharyngeal swabs were collected, one set by a lab technician and the other by the patients, and the COVID-19 rRT-PCR test was performed.

**Results:**

Of 50 selected cases, in seven patients all collected naso- and oropharyngeal swabs tested positive, and in 22 patients all samples tested negative for COVID-19 in rRT-PCR. Discrepancies between rRT-PCR results of lab technician- and patient-collected swabs were observed in 12 nasopharyngeal and 13 oropharyngeal specimens. Positive lab technician-collected and negative patient-collected samples were observed in 10 and 5 nasopharyngeal and oropharyngeal specimens, respectively. Negative lab technician-collected and positive patient-collected samples were observed in two and seven nasopharyngeal and oropharyngeal specimens, respectively. The overall percentage of agreement among both nasopharyngeal and oropharyngeal swabs taken by a lab technician and patients was 76% with a kappa value of 0.49 (*P*=0.001).

**Conclusion:**

Based on our findings, lab technician-collected naso- and oropharyngeal swabs cannot be replaced by patient-collected ones with regard to COVID-19 rRT-PCR.

## Introduction

Severe acute respiratory syndrome coronavirus 2 (SARS-CoV-2) was discovered in Hubei Province, China, in December 2019 for the first time and the World Health Organization (WHO) warned about a coronavirus disease 2019 (COVID-19) pandemic eventually (1,2). 

COVID-19 patients are asymptomatic or present with flu-like symptoms. The patient can only experience mild symptoms such as fever, dry cough, shortness of breath, fatigue, slight dyspnea, sore throat, headache, conjunctivitis, and gastrointestinal symptoms. However, some high-risk individuals can develop rapidly progressive acute respiratory distress syndrome (ARDS) (3, 4).

SARS-CoV-2 is highly transmissible and can be easily passed from human to human through droplets, contact, and fomites and indirectly by contact with contaminated environmental surfaces followed by self-inoculation of ocular, nasal or oral mucosa (4).

 COVID-19 can spread rapidly amongpatients and healthcare workers in healthcare settings and may have serious and severe complications in high-risk populations, particularly in the elderly, the critically ill, and the immunocompromised patients and young children (5).

A simple approach to prevent close contact in healthcare settings during the recent COVID-19 outbreak is to train patients to collect their own nasopharyngeal and oropharyngeal swabs and deliver them to medical laboratories to have them processed.

There are several beneﬁts to a home-testing sample collection method, including wider availability with lower costs and decreased risk of exposure to the virus. Home testing would also decentralize care and provide a wider area of coverage, particularly for older adults who have been identiﬁed as high-risk individuals due to a higher mortality rate in adults over 50 years of age (6).

The aim of our study was to determine the degree of concordance between naso- and oropharyngeal samples collected by the patient and a lab technician, using a real-time reverse transcription–polymerase chain reaction (rRT-PCR) assay for SARS-CoV-2.

##  Materials and Methods

The patients who participated in this study were selected from adults who had been referred to Imam Khomeini Hospital Complex (IKHC), Tehran, Iran, from February 28 to April 27 of 2020 with flu-like symptoms. According to the Center for Disease Control and Prevention (CDC), the flu-like symptoms are defined as cough and shortness of breath or difficulty in breathing accompanied by other symptoms such as fever, chills, muscle pain, sore throat, or new loss of taste or smell (7). We recruited all the patients who had undergone chest CT-Scan and had demonstrated radiographic features of atypical pneumonia (highly suggestive of COVID-19). All the patients were admitted to the infectious diseases ward of IKHC. Written instructions on how to collect the two required specimens were provided for the patients who participated in this study. The first two naso- and oropharyngeal samples were collected by the patients (after reading the instructions) and sent immediately to a clinical laboratory. The following two naso- and oropharyngeal samples were collected (the nasopharyngeal specimen was taken from the opposite nostril) by the lab technician. Specimens were obtained by a sterile Dacron swab with a plastic handle and soon after collection, each swab was placed into a separate container containing viral transport medium (VTM). The specimens were transported to a clinical laboratory within 30 minutes of collection and were processed soon after delivery. Patients’ demographic data and their clinical history were collected from their files. None of the patients had a history of having an occupation as a healthcare worker.

**COVID-19 Rapid Real-Time PCR**

*Total RNA Extraction*

In the present study, RNA extraction was performed using the Viral Nucleic Acid Extraction kit (Cat. No. YVN50/YVN100) provided by RBC Bioscience, Taipei, Taiwan. The process was initiated by transferring 200µL of the sample into a microcentrifuge tube. Then, 400µL of VB buffer in addition to 10µL of Proteinase K were added to the tube and the tube was incubated at 65°C for 10 minutes. By adding 500 µL of 95% ethanol and multiple rounds of washing, using W1 buffer and R-Wash buffer, centrifugation, and following use of 50µL RNase-free water and another round of centrifugation, eluted nucleic acid was extracted.

*Rapid real-time PCR*

This step was performed using the Novel Coronavirus (2019-nCOV) Nucleic Acid Diagnostic Kit (PCR-Fluorescence Probing) of Sansure Biotech (S3102E) (Changsha, China) and a thermal cycler specified for CFX96™ Real-Time PCR Detection System (Bio-Rad Laboratories, Inc.) by adding 30 µL PCR-Mastermix (including 2019-nCoV-PCR Mix and 2019-nCoV-PCR-Enzyme Mix, containing premiers, probes, dNTPs, MgCl2, Rnasin and PCR buffer for the 2019-nCoV-PCR Mix and RT enzyme and Taq enzyme for the 2019-nCoV-PCR-Enzyme Mix) into PCR reaction tube with 20 µL of the extracted RNA sample. Then, the specimen, 2019-nCOV-PCR positive control, and 2019-nCOV-PCR negative control were placed into the specimen wells of the amplification equipment. The first two steps “reverse transcription” and “cDNA predenaturation” were performed at 50°C (30 min) and 95°C (1 min), each with a single cycle, respectively. A 15-second cycle at 95°C, followed by a 30-second cycle at 60°C with 45 repetitions were performed. Then, the device cooled down to 25°C for 10 seconds to finalize the process. The device’s software was used for calculation of the cycle threshold (Ct) values. For 2019-nCoV-PCR negative control Ct value > 40 and for positive control Ct value No≤ 35 were defined based on each kit’s instructions. The RT-PCR results were reported as positive or negative. The whole PCR testing was done by technicians who were blind to the type of the collected specimen.

The research protocol was confirmed by the Ethics Committee of Tehran University of Medical Sciences (IR.TUMS.MEDICINE.REC.1399.048).

**Statistical Analyses:**

To identify the presence of a systematic difference between lab technician- and patient-collected sample results we used McNemar Chi-Square test and for assessing the concordance between test results we calculated Cohen's kappa value. A P-value of less than 0.05 was considered as statistically significant. The data were analyzed using SPSS 25 (SPSS Inc., Chicago, IL, USA). 

## Results

**Population Demographical Data:**

Fifty patients with clinical and radiologic evidence of viral pneumonia were entered into the study. All patients were admitted to the infectious diseases ward of IKHC. Thirty-two (64%) patients were male and 18 (36%) were female. The median age of participants was 56 years, ranging from 24 to 86 years. Among patients, fever (68%), muscle pain, and cough (60% each) were the most common chief complaints. The other symptoms are sorted in Table 1. Hypertension (9 out of 50 patients), cardiovascular diseases, diabetes mellitus, and respiratory illnesses (each 6 out of 50 patients) were the most notable underlying diseases (Table 2). Four patients had a history of antibiotic therapy (two with Azithromycin alone, one with Ciprofloxacin and Azithromycin and one patient did not remember the name of antibiotic used). The patients had neither a history of prior hospital admission nor a history of PCR testing for their current disease. O_2_ saturation levels which were measured by pulse oximeter were ≥93% in 30% of patients and <93% for 70% of the patients when coming to the hospital for the first time.

**Table 1:** Chief complaint of the patients suspected for COVID-19

Clinical symptoms	Fever	Muscle pain	Cough	Dyspnea	chills	Chest pain	Loss of taste or/and smell	sneeze	Abdominal symptoms
**Number and percentage of the patients**	34 (68%)	30 (60%)	30 (60%)	23 (46%)	8 (16%)	5 (10%)	4 (8%)	2 (4%)	1 (2%)

**Table 2:** Underlying medical conditions in patients underwent COVID-19 real-time PCR test

Associated medical conditions	Number of patients
**Hypertension**	9
**Cardiac problems**	6
**Diabetes**	6
**Respiratory problems**	6
**History of tuberculosis**	1
**Metastatic gastric cancer to lung**	1
**AIDS**	1
**Chronic liver disease**	1
**Hyperthyroidism**	1
**Pregnancy**	1

In this study, each patient had two sets of collected samples from nasopharyngeal and oropharyngeal swabs, one of each taken by patients their own and the other by a lab technician. Assuming patients with at least one swab showing a positive result as being infected with SARS-CoV-2, 26 out of 50 patients were infected. All patients with a double negative result for lab technician-collected samples showed a double negative result in patient-collected samples, too. The Chi-Square Tests table showed a McNemar P-value of 0.039 for nasopharyngeal samples and 0.77 for oropharyngeal samples (Table 3). The overall percentage of agreement among nasopharyngeal swabs taken by a lab technician and patients was 76% with a kappa value of 0.49 (*P*<0.001, 95% CI: 0.26-0.72) and the overall percentage of agreement among oropharyngeal swabs taken by a lab technician and patients was 76% with a kappa value of 0.49 (*P*=0.001, 95% CI: 0.24-0.74), after taking chance agreement into account (Table 4). Seven out of 50 patients had four positive PCR results for COVID-19 and 24 patients had four negative PCR results. Twelve patients were positive for both nasopharyngeal samples (taken by the patient and lab technician) and thirteen were positive for both oropharyngeal samples. Double negative nasopharyngeal PCR results (taken by patient and lab technician) were observed in 26 patients and double negative oropharyngeal PCR results were seen in 25 patients. Of remaining, ten patients had positive nasopharyngeal samples collected by the lab technician while their self-collected samples showed negative results and two patients had positive results for their self-collected nasopharyngeal specimen whereas the lab technician-collected specimen was negative for PCR testing. For oropharyngeal specimens, this discordance also existed. Five oropharyngial samples, taken by the lab technician had positive PCR results while their self-collected samples were negative and seven self-collected oropharyngeal specimens were positive for COVID-19 PCR with negative results for lab technician-collected specimens. In four patients, lab technician-collected samples were positive (two for both samples and two for one of naso- or oropharyngeal samples) whereas their self-collected samples showed negative PCR results.

**Table 3:** Nasopharyngeal and oropharyngeal swabs collected for the diagnosis of COVID-19 using rRT-PCR by the lab technician and patients

	Percentage of positive results in patient-collected samples	Percentage of positive results in lab technician-collected samples	Proportion in agreement	Kappa 95% CI	P-value
**Nasopharyngeal swab**	28%	44%	0.76	0.49 (0.26, 0.72)	<0.001
**Oropharyngeal swab**	36%	40%	0.76	0.49 (0.24, 0.74)	=0.001

**Table 4:** Comparative results of novel coronavirus detected in lab technician- and patient-collected naso- and oropharyngeal swabs

Nasopharyngeal specimens		Lab technician-collected		Total	Oropharyngeal specimens		Lab technician-collected		Total
		-	+				-	+	
**Collected by the Patients **	-	26	10	36	Patient collected	-	25	7	32
	+	2	12	14		+	5	13	18
**Total**		28	22	50	Total		30	20	50
**P-value=0.039**					P-value=0.77				

## Discussion

The aim of our study was to determine whether the rRT-PCR results were influenced if the nasopharyngeal and oropharyngeal samples were collected by lab technicians or patients. Our findings about the nasopharyngeal samples showed a systematic difference as well as a moderate agreement between the two. In case of the oropharyngeal samples, although there is no systematic difference between these two collection methods, a moderate agreement (kappa value = 049) was observed between them. Accordingly, in neither of these cases, patient-collected samples can replace the samples collected by lab technicians. The positive tests were more observed in lab technician-collected samples and discordant results between naso- and oropharyngeal samples were more found in self-collected samples (12 patients for self-collected samples versus 10 for lab technician-collected samples). COVID-19 is a new disease and research and experience about self-collected nasal swabs are limited (7-10). However, several studies in pediatrics’ field have shown that parental-collected midturbinate, nasopharyngeal, and/or throat specimens for detecting viruses such as human meta pneumovirus, influenza A virus, influenza B virus, respiratory syncytial virus, parainfluenza viruses, and adenoviruses is an efficient and acceptable method (11-12).

Neelam Dhiman *et al.* demonstrated that health care worker (HCW)- and patient-collected nasal swabs, using flocked nasal midturbinate swab, were suitable alternatives for the detection of influenza A and B using rRT-PCR test. There was no significant difference in the overall positivity rate by either collection methods, and self-collection method was well accepted by patients. Of the 72 paired specimens analyzed, 34.7% were positive for influenza A or B RNA by at least one of the collection methods. When the 14 patients who had prior health care training were excluded, the qualitative agreement between collection methods was 94.8% (55 of 58). A total of 53.4% of patients (31 of 58) preferred the self-collection method over the HCW collection, and 25.9% (15 of 58) had no preference (5).

Esposito *et al.* directly compared parent-collected midturbinate nasal swabs with pediatrician-collected swabs for influenza detection by rRT-PCR and demonstrated moderately high sensitivity (89.3%) and specificity (97.7%) for the parental collection technique. They also demonstrated that the direct involvement of parents in the collection process increased the child’s acceptance of the sample collection (10).

In a study by Christopher P. Seaman, it was suggested that self-collection is highly comparable to professional-collection for diagnosis of influenza in symptomatic individuals, and they have demonstrated that findings from studies using self-collection are probably only minimally affected by measurement errors. They identified 14 studies that compared the diagnostic accuracy of self-collected to professional-collected swabs in symptomatic infected individuals by influenza. Self-collected swabs were found to be highly acceptable, simple, and comfortable to use. Data from nine studies were meta-analyzed. Pooled sensitivity was 87% (95% CI: 80%-92%) and specificity was 99% (95% CI: 98%-100%), compared to professional-collected swabs in the diagnosis of influenza. Pooled sensitivity and specificity estimates were used to assess the potential bias that would be introduced in studies that used the self-collected samples rather than professional collected ones. While self-collected swabbing should not replace the role of clinical testing, findings support the use of self-collected swabs for influenza research and surveillance (11). 

In Michael L Jackson’s study, a self-collected nasal swab for respiratory virus surveillance was evaluated. They tested 135 patients with acute respiratory illness (ARI) who could self-collect nasal swab specimens and send them for laboratory testing. Most patients (78.2%) collected and shipped their specimens without errors. These results support the use of self-collected nasal swabs in community-based respiratory virus studies. In this study, subjects with ARI were enrolled by phone, with no opportunity for in-person training in the study procedures. The similar prevalence of viruses in the self-collected specimens versus clinical specimens from the same period suggests that sensitivity is sufﬁciently high for most research purposes (11).

Nasal swab testing for COVID19 has recently been assessed with emerging data on the performance of the available RT-PCR tests (13). Self-testing for viral respiratory illnesses itself is not new and has been described in inﬂuenza, where there is much more experience (14-17). 

In another study, participants were asked to self-collect swabs in an emergency department. Those swabs were compared with swabs collected by health care professionals in the opposite nostril. Results were comparable; 90% of participants found self-collection to be easy or very easy, and only 21% preferred health care professional collection versus self-collection. Notably, a self-testing strategy would be oﬀered only at the direction of a clinician with an understanding that no test is perfect, much like many of our available tests for other illnesses (5).

Shantanu Nudy *et al.* suggested that expanding access to a self-service diagnostic pathway for COVID19 would be beneficial, by utilizing at-home nasopharyngeal swab collections and telemedicine services, with the help of a qualiﬁed clinician who would triage the patients and determine whether testing is appropriate or not, based on the latest CDC guidelines. Individuals who are able to be safely tested at home would receive a test kit through home delivery from a local distribution site; a pickup at a local clinic, pharmacy, or public health center; or by mail; and then swab their nasopharynx themselves or with the help of caregivers (6).

Although due to these studies self-collection is assumed to be an efficient and useful method of sample collection for viral diseases such as influenza A, influenza B, RSV, and adenovirus, for the new pathogen COVID-19 the same may not be true. Much less is known about its pathogenesis and its probable behavioral changes are under study. Therefore, according to our study, self-collection methods cannot be simply applied for COVID-19 patients. In our study, 50 patients of a wide age range, some of whom had underlying diseases, participated in the research. The small sample size may be considered as a limitation of our study. Other factors such as a wide age range and present comorbidities may also affect the results. Hence studies with a larger sample size that take the age group and associated diseases into account could alter the final results. 

The inclusion of a written instruction for participants about sample collection could enhance their technique and ability of proper sampling. In our study, of four patients with at least one positive lab technician-collected sample who showed negative result in both self-collected ones, three of them were 60-year-old or more, so it seems that more attention and caution are necessitated for older age groups and additional teaching and practicing should be dedicated to them for more accurate results. In this age group, eleven patients had a history of underlying diseases such as diabetes, hypertension, ischemic heart disease, and respiratory problems. Nine out of the eleven patients had O_2_ saturation levels of less than 93%. Thus, self-collection sampling is not recommended for these patients with severe medical conditions and serious medical treatment and care should be initiated immediately even if the laboratory results are negative. So as Neelam Dhiman’s study explained, “personalized patient care” is the preferred measure (5).

Prior antibiotic therapy is also an important issue (18). In our study three out of four patients with a history of antibiotic therapy showed four negative PCR results. Thus, it seems to be crucial to separate patients with a history of antibiotic therapy and to study them as a distinct patient group.

Currently, CDC recommends PCR testing for the detection of COVID-19. In a study by Chunqin Long *et al.*, sensitivity of CT scan and rRT-PCR were compared. Based on their study, a CT scan had a sensitivity of 97.2%, whereas the sensitivity of initial rRT-PCR was only 83.3% (14). CT Scan has a higher sensitivity and a lower false negative rate compared to PCR (15). In our study, 24 out of 50 (48%) patients with clinical and radiological evidence of COVID-19 had four negative PCR results. So as stated above, PCR testing alone can lead to false negative results and is not able to detect COVID-19 (16,19-22). CDC proposes the superiority of PCR testing in the first week after infection because it takes 1-3 weeks for the body to make antibodies and after that period, serologic tests are preferred (23-26). Therefore, a combination of PCR and serologic tests are the best laboratory diagnostic approach to detection of COVID-19 (27-29). Special attention should be paid to the clinical course and the radiological examination of all suspected cases as soon as possible.

In specimens obtained from clinically and radiologically infected patients, 22 nasopharyngeal lab technician-collected samples showed positive results, whereas only 14 self-collected samples were positive and 20 oropharyngeal lab technician-collected sample were positive compared to 18 positive results in patient-collected ones. This demonstrates a better sampling technique by lab technicians more obviously in nasopharyngeal swab. Overall, considering positive results for lab technician and self-collected samples for both naso- and oropharyngeal specimens, lab technicians showed a better performance in sample collection.

## Conclusion

Our study showed a moderate agreement between the specimens collected by the patients and those collected by the lab technicians in both nasopharyngeal and oropharyngeal samples therefore, in COVID-19 cases, sample collection by lab technicians as the reference method has priority over sample collection by patients and should not be replaced by the latter.
